# Crohn’s disease in hemophilic arthropathy patient: a case report

**DOI:** 10.1186/s12891-024-07610-y

**Published:** 2024-06-28

**Authors:** Zhongyi Zhang, Lei Chen, Haojing Zhou, Guoqian Chen, Peijian Tong

**Affiliations:** The First Affiliated Hospital of Zhejiang Chinese Medical University (Zhejiang Provincial Hospital of Chinese Medicine), Hangzhou, 310006 China

**Keywords:** Crohn’s disease, Hemophilic arthropathy, Genetic testing, Nutritional intervention

## Abstract

Crohn’s disease (CD) is an inflammatory bowel disease affecting the digestive tract, the incidence of which is on the rise worldwide. The most common clinical manifestation of hemophilia is arthropathy secondary to recurrent joint effusions and chronic synovitis. This article reports on a rare 25-year-old male patient with both hemophilic arthropathy and Crohn’s disease who was at risk for pathogenic gastrointestinal bleeding. After undergoing endoscopic pathologic testing and genetic testing, a multidisciplinary expert work-up of a treatment and nutritional plan was performed. The patient improved clinically and adhered to conservative treatment. This case report is the first report of this rare co-morbidity, demonstrating the highly pathogenic mutation locus and summarizing the clinical experience of early diagnosis and treatment.

## Introduction

Crohn’s disease (CD) constitutes a chronic affliction of the gastrointestinal tract, while ulcerative colitis (UC) figures prominently among the principal varieties of inflammatory bowel disease (IBD) [1]. The CD is notable for its potential to involve any segment of the digestive system, manifesting predominantly through symptoms such as gastrointestinal haemorrhage, abdominal discomfort, diarrhoea, weight reduction, and the presence of abdominal masses [2]. Treatment options include steroids, immunosuppressants (such as azathioprine and methotrexate), biologics (such as infliximab, adalimumab, ustekinumab, vedolizumab, and golimumab), and nutritional support, with surgical intervention when necessary [3]. The aetiology of CD remains elusive; however, prevailing hypotheses implicate an aberrant immunological response, with genetic predispositions, environmental triggers, and immunological factors contributing to disease pathogenesis [4]. The global incidence of CD is on an upward trajectory. Diagnostic confirmation of CD relies on colonoscopic examination and histopathological evaluation, with optimal management aiming to mitigate recurrence risk, halt disease propagation, and obviate the need for immunosuppressive therapy [5].

Conversely, Hemophilia A is an X-linked recessive genetic disorder characterized by a deficiency in coagulation Factor VIII (F),resulting in impaired hemostasis [6]. Depending on the type of coagulation factor the patient is deficient in, haemophilia is classified as haemophilia A and haemophilia B. The severity of the disease is classified according to the results of the measurement of factor VIII (FVIII) and factor FIX (FIX) activity [7].The prevalence rates for Hemophilia A and B stand at 17.1 per 100,000 male births, with an estimated global hemophilia population of 1.125 million, of which 418,000 cases are classified as severe. The hallmark of hemophilia is a propensity for bleeding, particularly in synovial joints such as the knees, ankles, and elbows [8]. Recurrent hemarthroses lead to hemophilic arthropathy in up to 90% of individuals by their third decade, manifesting as joint pain and deformity, markedly diminishing life quality. Management primarily involves F replacement therapy and prophylactic measures against bleeding episodes, with diagnosis confirmed through coagulation factor assays [9]. The concurrence of hemophilic arthropathy and CD in a patient with Hemophilia A is exceedingly rare, given their distinct etiological bases and clinical triggers.

This case report delineates the clinical journey of a patient grappling with both conditions, emphasizing the diagnostic process, therapeutic interventions, and the multifaceted challenges encountered in concurrent management. Furthermore, this report seeks to elucidate potential genetic correlations between the two conditions, thereby enriching our comprehension of this unusual clinical confluence. Through an in-depth exploration of this case, we aim to augment the existing body of knowledge concerning the intricacies of managing hemophilic arthropathy and CD, thereby offering invaluable insights for future clinical endeavours.

### Aim

Reporting a case of Crohn’s disease complicated by hemophilic arthropathy, with the recent diagnosis of Crohn’s disease in a patient previously diagnosed with hemophilic arthropathy.

### Case report

A 25-year-old Asian male, diagnosed with moderate Hemophilia A (with a Factor VIII activity level at 4.8% and absence of Factor VIII inhibitors), sought medical attention at our institution due to persistent right knee discomfort extending over a year. The patient’s medical history was notable for recurrent, uncontrolled bleeding episodes and epistaxis from childhood following minor injuries, culminating in a diagnosis of Hemophilia A. His therapeutic regimen included thrice-weekly intravenous infusions of Factor VIII, dosed at 2000 IU per session. Upon presentation to the orthopaedic division, the predominant symptom articulated was enduring right knee pain, spanning more than a year. Initial assessment revealed stable vital signs (temperature at 36.5 °C, respiratory rate of 18/min, heart rate at 87 beats/min, and blood pressure recorded at 129/74 mmHg). The patient negated any antecedent traumatic incidents, drug allergies, or a medical history suggestive of infectious conditions, including tuberculosis.

**Fig. 1:**
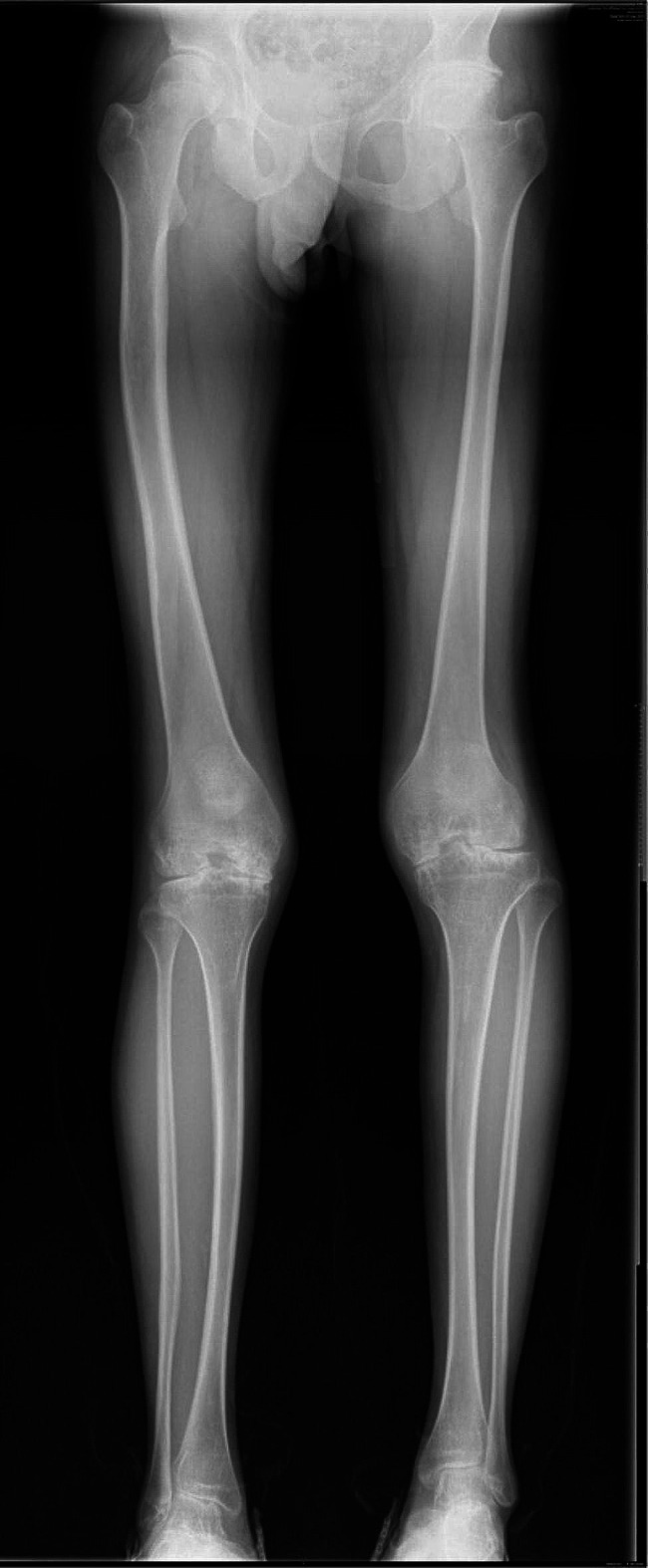
Full-length X-rays of both lower limbs showed a significant narrowing of joint spaces, severe sclerotic changes, and pronounced deformities

In the case where the patient’s history of hemophilia is clear, we found that their knee osteoarthritis reached stage IV according to the Kellgren-Lawrence (KL) grading system (Fig. 1 full-length radiographs of both lower limbs), with limb swelling, stiffness, and limited mobility. The condition has reached the surgical indication for total knee arthroplasty (TKA). We invited a senior hematologist for consultation to adjust the treatment plan, including coagulation factor therapy, and planned to proceed with TKA after relevant preoperative examinations and normalization of coagulation function. We conducted a detailed assessment using the Western Ontario and McMaster Universities Osteoarthritis Index (WOMAC) and Knee Society Score (KSS), scoring 22 and 126 points respectively, indicating significant limitations in daily activities. Subsequent researchers provided gait analysis, with the following results: stride length 1.09 ± 0.02, stride width 0.16 ± 0.01 m, right (step length 0.55 ± 0.01 m, initial double limb support 0.11 ± 0.01 s, gait profile score 7.6, hip flexion 6.0, knee flexion 9.7), left (step length 0.54 ± 0.02 m, initial double limb support 0.10 ± 0.01 s, gait profile score 9.2, hip flexion 4.4, knee flexion 9.0). Based on the gait analysis, it can be inferred that the patient’s gait differs significantly from that of a normal individual, especially in terms of stride, step length, and hip and knee joint extension of the lower limbs on both sides.

Three days after admission, we gave coagulation factor replacement therapy, Enteral Nutritional Powder to improve enteral nutrition, together with traditional Chinese medicine internal and external treatment, the patient’s joint pain was relieved, inflammation was reduced, and he asked for a temporary deferral of surgery on his own.

**Fig. 2:**
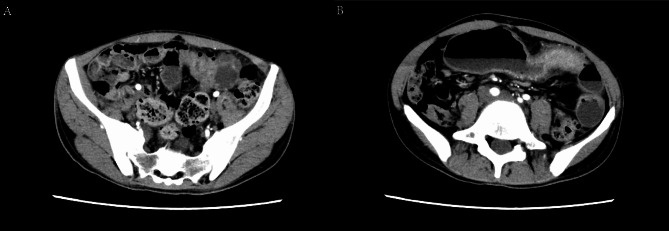
Abdominal enhanced CT: Group 3–4 small intestine with localised significant thickening of the intestinal wall with localised narrowing of the intestinal lumen, and significant dilatation of the proximal intestinal tubes with gas-liquid planes

The patient presented to our gastroenterology department due to “intermittent melena for 2 weeks” with accompanying abdominal distension. Blood tests revealed hemoglobin of 59 g/L and a platelet count of 188*10^9/L. A preliminary diagnosis of intestinal proliferative changes with lymphatic dilation was made. The patient had a history of anemia for several years with a history of blood transfusion but no transfusion reactions. Anorectal examination revealed internal and external hemorrhoids. Abdominal CT scan (Fig. 2A: Group 3–4 small bowel with significant localised bowel wall thickening with localised bowel lumen narrowing; B: Enhancement showing significant air-liquid planes)showed localized thickening of the small intestinal wall with focal luminal narrowing, suggesting partial intestinal obstruction, and indicating a possibility of inflammatory bowel disease (in the active phase).

**Fig. 3:**
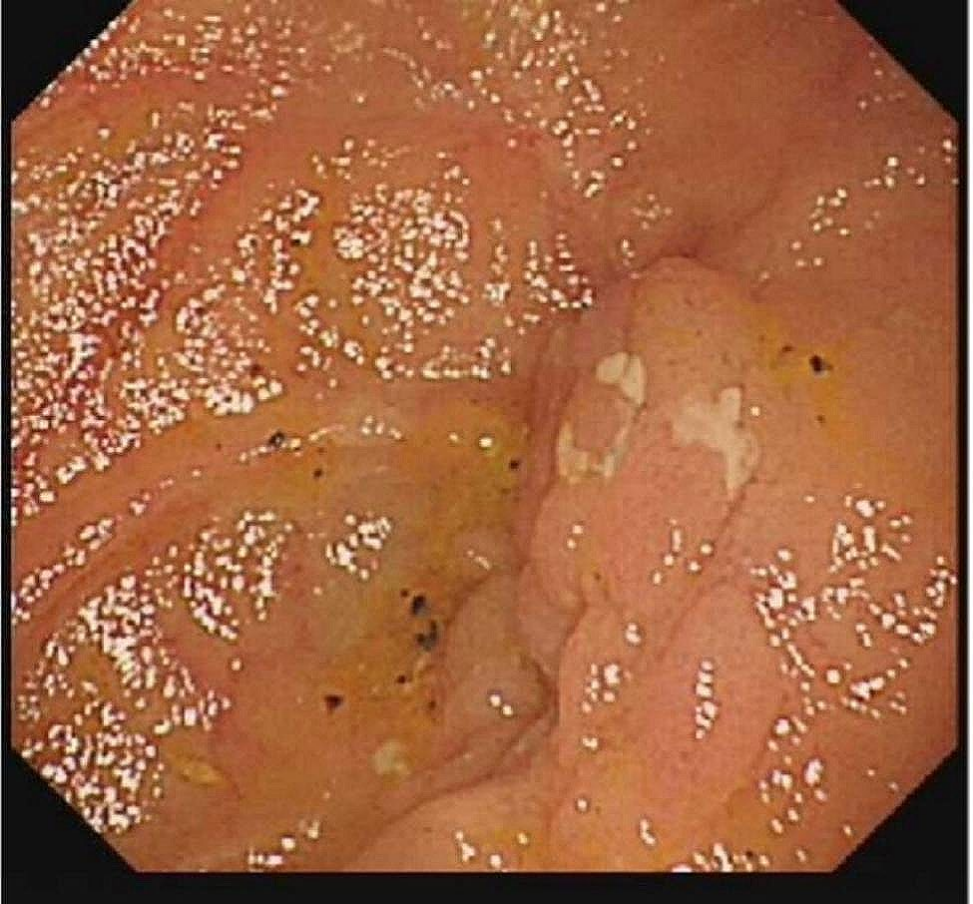
Transoral small bowel microscopy after factor VIII injection, reaching group 2 small bowel, reveals peripheral stenosis with ulceration

**Fig. 4:**
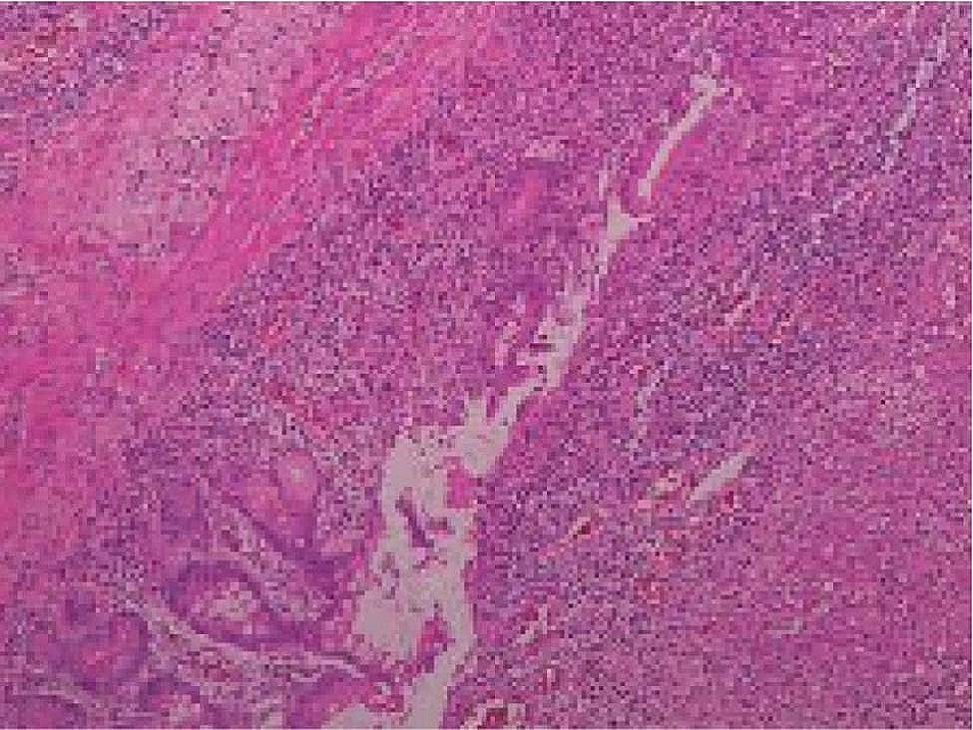
Hematoxylin-eosin stain result: Ki-67(Scattered +), P53(Occasional +), CD68(Tissue cells +), CD163(Tissue cells +), S-100, (Nerve+), MUC-2(+), MUC-6(zones +)

The patient underwent painless endoscopy and capsule endoscopy(Figure 3 Transoral small bowel microscopy after factor VIII injection, reaching group 2 small bowel, reveals peripheral stenosis with ulceration). After receiving factor VIII transfusion, enteroscopy was performed. Approximately 20 min into the procedure, small intestinal dilation was observed with circumferential narrowing below, without ulcers. The endoscope could not pass through, and three biopsies were taken, resulting in minimal oozing but no active bleeding. The results of this report showed jejunal stenosis with ulceration, considering inflammatory conditions. Combining the above findings and the doctor’s assessment, a partial small bowel.Subsequent laparoscopic exploration revealed partial small bowel resection. Partial small bowel resection specimen showed, on gross examination: the mesenteric portion of the small bowel was creeping towards the sides of the bowel, and longitudinal fissure-like ulcers were visible on the mucosal surface of the mesenteric side; Pathological examination confirmed Crohn’s disease, showing moderate chronic active inflammation of the small intestine mucosa. Postoperatively, the patient received standard mesalazine treatment, which was later switched to ustekinumab due to lack of efficacy and adverse gastrointestinal reactions. The initial dose was 260 mg followed by subcutaneous injections of 90 mg. Subsequent routine colonoscopies for Crohn’s disease revealed A2 L4 B2, indicating moderate remission (Fig. 4 Immunohistochemical staining result).

In addition, we sought the patient’s opinion and conducted whole exome genetic testing on them. The specific experimental method was as follows: The total DNA was extracted using a standard DNA extraction protocol. Then, the DNA, which was fragmented using sonication, was subjected to library construction. Exome capture was performed using the SureSelect Human All Exon V6 Kit (Agilent Technologies) following the vendor’s recommended protocol. The sequencing was carried out using the Illumina Novaseq™ 6000 with 150-bp paired-end sequencing mode. Our research team screened through 30,272 types of mutations using basic databases, mutation frequency databases, deleterious software scoring, and phenotype databases, combined with our own knowledge base. We identified three clinically significant pathogenicmutations: NM_020937.2:c.4931G > A,NM_001458.5:c.7614G > T;NC_000006.11:g.152583258T > A. Based on our second-generation sequencing, we may follow up with one-generation sequencing to further identify disease-causing mutations. Currently, the patient is under ongoing follow-up and monitoring.

## Discussion

This case report explains the conundrum posed by a patient grappling with the dual diagnoses of hemophilic arthropathy and Crohn’s Disease (CD), a juxtaposition scarcely documented in medical literature. Firstly, it demonstrates a complex spectrum of diseases confirmed through small intestinal biopsy, genetic testing, orthopedic examination and scoring. In our clinical practice, managing either hemophilic arthropathy or Crohn’s disease alone presents a significant challenge for physicians. Probably contrary to most people’s suspicions, hemophilia maybe can protect against CD through various mechanisms [10], which based on platelet levels, fibrinogen and other factors, such as the common occurrence of portal vein, mesenteric vein thrombosis, or cerebral sinus thrombosis in patients with inflammatory bowel disease [11–14]. However, due to coagulation disorders in hemophilia patients, inflammation and bleeding can also exacerbate. Therefore, the exact mechanism of CD occurrence in hemophilic arthropathy patients is unclear, possibly due to impaired local intestinal thrombosis function, with acquired hemostatic changes possibly playing an important role during active disease. Inflammation can activate the coagulation cascade, promote platelet aggregation, and impair anticoagulant or fibrinolytic mechanisms, leading to a hypercoagulable state [15, 16]. The incidence of CD in hemophilia patients is significantly lower, resulting in a lack of information on the disease’s combination [17]. Patients may present with easy bruising, trauma, or minor injuries due to coagulation deficiency, or spontaneous bleeding in severe hemophilia patients. Secondly, the treatment regimen is unclear. The patient has reached the surgical indication but requests conservative treatment. Further research is needed for perioperative bleeding management. Thirdly, there are no direct case reports and opinions, except for a few reports and opinions on hemophilia complicated by ulcerative colitis and duodenal hematoma [18–23]. There is almost no data on concurrent hemophilia A and Crohn’s disease. Therefore, there is no evidence-based scheme besides recommending treatment or prevention for each situation separately to prevent further episodes. Early diagnosis, prevention of CD exacerbations, and the use of factor VIII and antihemorrhagic drugs are crucial for preventing disease progression.

We identified three clinically significant pathogenic mutations: NM_020937.2:c.4931G > A, clinically significant as it can lead to Fanconi anemia; NM_001458.5:c.7614G > T, causing cardiomyopathy and muscular atrophy; NC_000006.11:g.152583258T > A, potentially resulting in muscular dystrophy or autosomal recessive inheritance. With the patient’s medical history known, all these mutations’ clinical manifestations were observed. Of particular interest is one mutation indicating autosomal recessive inheritance, while hemophilia is an X-linked recessive genetic disorder. This report may provide genetic evidence for patients with hemophilic arthropathy and Crohn’s disease, offering valuable insights for subsequent diagnosis and treatment. As this assay used high-throughput second-generation sequencing, the results obtained were subject to a high degree of error, and subsequent one-generation sequencing of highly pathogenic mutations was proposed. Relevant literature [24] found that Three loci (NOD2, MHC, and MST1 3p21) were associated with sub phenotypes of inflammatory bowel disease, mainly disease location (essentially fixed over time; median follow-up of 10·5 years). In the field of hemophilia, there is a plethora of genetic research components, and the severity of the disease depends on the reduction in the level of coagulation FVIII or FIX activity, which depends on the type of causative variant in the genes encoding the two factors (F8 and F9, respectively) [25]. It is evident that enhanced genetic testing and early diagnosis of hemophilia and Crohn’s disease greatly optimizes patient care.

In this case report, our team of hematologists, gastroenterologists and orthopaedic surgeons work together to develop and manage the patient’s treatment and nutritional plan. In such rare comorbid conditions, multidisciplinary discussions are essential for developing the.

patient’s treatment and VTE management plan, including nursing methods, surgical interventions, and nutritional support. This not only facilitates the implementation of personalized treatment plans but also foresees significant improvements in the patient’s prognosis, including long-term disease control and quality of life enhancement.

Nutritional intervention and dietary strategies have been explored as adjunctive therapies for IBD [26]. Specific enteral nutrition can effectively induce remission in Crohn’s disease. The focus of dietary strategies is to adjust the consumption ratio of pro-inflammatory or anti-inflammatory nutrients [27]. Additionally, nutritional strategies and dietary therapy play a crucial role in Crohn’s disease treatment due to their availability, low cost, and minimal side effects. Diet can influence the structure and function of the intestinal microbiota [28], thereby directly and indirectly affecting immune function. Furthermore, diet and nutrient composition can alter the permeability of the mucosal barrier. The patient underwent enteral nutrition (Ensure) therapy at our hospital, with a daily oral intake of 800 g as a nutritional supplement. Given the combination of hemophilia and Crohn’s disease, enteral nutrition alone appears insufficient, and dietary supplements can include specific supplements (such as vitamin D, omega-3 fatty acids) and probiotic supplements. Existing literature indicates that composite probiotics have a positive effect on Crohn’s disease [29]. How to integrate special nutritional strategies for hemophilia is a question that urgently needs to be addressed.

This case illustrates that without multidisciplinary management and treatment, patients with hemophilic arthropathy accompanied by CD may lead to severe and potentially fatal bleeding in later stages, exacerbating bleeding within the joints. Utilizing welfare medical insurance policies and multidisciplinary consultations for the treatment of these two comorbidities is the safest management strategy. Subsequent prevention of sudden bleeding events in CD and late-stage activity disorders in hemophilic arthropathy through biological agents and FVIII therapy requires complex health monitoring and follow-up visits combined with patient compliance.

## Conclusion

This case emphasizes the treatment challenges and risks of managing hemophilic arthropathy complicated by Crohn’s disease and, in conjunction with genetic testing, explore the genetic correlation between the two conditions to develop rational treatment and management strategies for such patients at the genetic level. The management of bleeding resulting from CD-induced gastrointestinal bleeding and intestinal obstruction, as well as joint cavity bleeding in hemophilic arthropathy, is crucial. Through early genetic testing, we hope to provide case support and genetic evidence for this rare yet significant comorbidity, enabling rapid screening, diagnosis, control of bleeding events, and formulation of nutritional support strategies.

## Data Availability

The datasets used and/or analyzed during the current study are available from the corresponding author on reasonable request.
